# Explainable machine learning for predicting childhood anemia in Sub-Saharan Africa using population-based DHS Data (2016–2024)

**DOI:** 10.1371/journal.pgph.0006999

**Published:** 2026-08-12

**Authors:** Andualem Enyew Gedefaw, Amanuel Worku, Abraham Keffale Mengistu, Bewuketu Terefe, Eliyas Addisu Taye, Fentahun Bikale Kebede, Nebebe Demis Baykemagn, Tirualem Zeleke Yehuala, Jamilu Sani

**Affiliations:** 1 Department of Health Informatics, Institute of Public Health, College of Medicine and Health Sciences, University of Gondar, Gondar, Ethiopia; 2 School of Information Science, Addis Ababa University, Addis Ababa, Ethiopia; 3 Department of Health Informatics, College of Medicine Health Science, Debre Markos University, Debre Markos, Ethiopia; 4 College of Medicine and Health Sciences, University of Gondar, Gondar, Ethiopia; 5 School of Public Health, The University of Queensland, Brisbane, Australia; 6 Strategic Affairs Executive Office, Federal Ministry of Health, Addis Ababa, Ethiopia; 7 Department of Demography and Social Statistics, Federal University Birnin Kebbi, Birnin Kebbi, Nigeria; VART Consulting PVT LTD, INDIA

## Abstract

Childhood anemia remains a major public health challenge in Sub-Saharan Africa, adversely affecting physical growth, cognitive development, and child survival. The pooled prevalence across 26 countries exceeds 60%, underscoring the need for accurate and scalable prediction tools to support targeted interventions.This study used pooled Demographic and Health Survey (DHS) data (2016–2024) from 26 Sub-Saharan African countries, including 110,251 children aged 6–59 months. Multiple machine learning models (Logistic Regression, Decision Tree, Extra Trees, Random Forest, XGBoost, LightGBM, CatBoost, and MLP) were trained using hyperparameter tuning with 5-fold cross-validation. Model performance was evaluated using accuracy, precision, recall, F1-score, and ROC-AUC, with 95% confidence intervals estimated via bootstrapping. Model comparisons were conducted using the DeLong test, and SHAP was used for model interpretability.The CatBoost model demonstrated the best overall performance (ROC-AUC = 0.84, 95% CI: 0.840–0.848), followed closely by XGBoost and LightGBM. All machine learning models significantly outperformed logistic regression (p < 0.001, DeLong test), although the absolute improvement in discrimination was modest (ΔAUC ≈ 0.04). The models demonstrated moderate discriminatory ability, with relatively low to moderate sensitivity (recall ≈ 0.33–0.41) depending on the model and classification threshold. SHAP analysis identified residence type, height-for-age z-score, country, and child age as the most influential predictors.Machine learning models demonstrated moderate to strong discriminatory performance in predicting childhood anemia using DHS data. Although improvements over traditional models were statistically significant, the relatively low sensitivity limits their effectiveness as standalone screening tools. These findings highlight the importance of early childhood nutrition (particularly during 6–23 months), reduction of chronic undernutrition, and context-specific public health strategies. Integration of predictive analytics into national health systems may support risk stratification and resource allocation in high-burden settings. However, findings should be interpreted in light of the cross-sectional design and lack of external validation.

## Introduction

Childhood anemia remains a significant public health problem in Sub-Saharan Africa (SSA), disproportionately affecting children under five years of age. Anemia is primarily characterized by reduced hemoglobin concentration, leading to impaired oxygen transport, which negatively impacts physical growth, cognitive development, and immune function. Globally, it is estimated that approximately 42% of children under five years of age are anemic, but in SSA, the prevalence often exceeds 40%, reaching levels as high as 60–70% in some countries [1,2]. The consequences of anemia in early childhood are profound, ranging from delayed psychomotor development to increased susceptibility to infection and higher mortality rates [3,4].

Despite ongoing interventions, including iron supplementation programs, deworming, and nutritional education campaigns, childhood anemia remains highly prevalent in Sub-Saharan Africa (SSA). Several factors contribute to the persistence of anemia, including chronic undernutrition, high rates of infectious diseases (e.g., malaria and helminthiasis), poor maternal health, inadequate sanitation, and socioeconomic disparities [5–8]. Importantly, the multifactorial etiology of anemia, encompassing nutritional deficiencies, infectious diseases, and genetic hemoglobin disorders, complicates effective intervention design and highlights the need for comprehensive approaches. Traditional epidemiological studies have examined these factors using regression-based methods; however, these approaches often struggle to capture complex interactions between multiple predictors and may underperform when applied to large, multi-country datasets [9].

While previous analyses of DHS and other nationally representative survey data have primarily relied on multilevel and logistic regression models to identify predictors of childhood anemia [10,11], only a limited number of studies have applied machine learning approaches to these complex, multi-country datasets. Existing ML-based studies have largely emphasized predictive discrimination rather than policy translation, model calibration, or the stability of key predictors across algorithms and country contexts. Consequently, there is currently no consensus on which ML models consistently perform best for childhood anemia prediction in population-based survey data, particularly when the primary objective is public health screening rather than statistical accuracy.

Machine learning (ML) provides an alternative approach capable of handling large-scale, high-dimensional datasets, identifying nonlinear relationships, and uncovering complex patterns that traditional statistical methods may overlook. Ensemble models, such as Random Forest (RF), Gradient Boosting Machines (GBM), and XGBoost, as well as neural network approaches, have been successfully applied to predict health outcomes, including malnutrition, infectious diseases, and anemia [12–14]. By integrating feature selection algorithms, such as interpretability tools such as SHAP (Shapley Additive exPlanations), ML models can identify the most influential predictors while offering actionable insights for policymakers [15,16].

Furthermore, ML approaches enhance predictive accuracy and enable the identification of critical windows for intervention, facilitating the development of targeted public health strategies in resource-limited settings.

Accordingly, this study pursues two complementary objectives: [1] to systematically compare the predictive performance, recall-oriented screening utility, and probability calibration of eight machine learning models applied to pooled DHS data, and [2] to generate interpretable, policy-relevant insights using explainable AI methods (SHAP) that can inform nutritional, maternal, and household-level interventions in diverse SSA contexts.

This study aims to leverage ML techniques to predict childhood anemia among children aged 6–59 months in 26 SSA countries using Demographic and Health Survey (DHS) data from 2016 to 2024. A total of 110,251 children were included in the analysis, of whom 41,685 had anemia. The study evaluated the predictive performance of eight ML models (random forest, Light Gradient Boosting Machine, CatBoost, XGBoost, Extra Trees, logistic regression, multi-layer perceptron neural network, and decision tree) and identified the most influential predictors of childhood anemia. By providing a robust, data-driven prediction framework, this study aims to inform early intervention strategies, optimize resource allocation, and guide public health policies tailored for SSA.

Previous research has demonstrated that anemia remains highly prevalent among children in SSA, with rates varying widely across countries due to differences in socioeconomic status, nutritional practices, and disease burden. For instance, DHS data analyses have reported an anemia prevalence of 61.9% among children across multiple SSA countries [17]. Malnutrition, particularly stunting (low height-for-age), has consistently emerged as a strong predictor of anemia, reflecting the critical link between chronic undernutrition and impaired hemoglobin synthesis [18]. Other studies have highlighted that younger children, especially those aged 6–23 months, are at a higher risk due to inadequate complementary feeding and increased iron requirements during rapid growth periods [19].

Maternal characteristics also play significant roles. Lower maternal age, low educational attainment, and poor nutritional status increase the likelihood of anemia in children [9]. Household factors, including wealth index, household size, and number of children under five years old, influence access to resources, nutrition, and healthcare, further affecting the prevalence of anemia [20]. Environmental predictors, such as access to improved sanitation and safe drinking water, have been linked to reduced infection risk and lower anemia rates [2].

## Methods and materials

### Ethics statement

This study was based on a secondary analysis of anonymized Demographic and Health Survey (DHS) data obtained from the DHS Program after formal approval to access the datasets. Ethical approval for the original surveys was obtained by the DHS Program and the relevant national ethics committees in each participating country. Written informed consent was obtained from all participants or their parents/guardians before data collection. Because this study involved analysis of publicly available, de-identified secondary data, additional institutional ethical approval and participant consent were not required.

### Study design and setting

This study employed a retrospective analysis of pooled cross-sectional data from the Demographic and Health Surveys (DHS) conducted between 2016 and 2024 in 26 Sub-Saharan African countries, including Angola, Benin, Burkina Faso, Burundi, Cameroon, Côte d’Ivoire, Ethiopia, Gabon, Gambia, Ghana, Guinea, Lesotho, Liberia, Madagascar, Malawi, Mali, Mauritania, Mozambique, Nigeria, Rwanda, Sierra Leone, South Africa, Tanzania, Uganda, Zambia, and Zimbabwe. The DHS is a nationally representative household survey that utilizes a stratified two-stage cluster sampling design to collect detailed information on child health, nutrition, maternal health, household characteristics, and socioeconomic factors.

To ensure valid cross-country inferences, all descriptive statistics and country-level prevalence estimates incorporated DHS sampling weights (v005), primary sampling units (v021), and stratification variables (v022/v023). Survey-weighted analyses were performed using the survey package in R and statsmodels. survey module in Python, allowing appropriate variance estimation under a complex sampling design.

However, for machine learning model development, unweighted data were used, as the primary objective was predictive modeling rather than population parameter estimation. This approach is consistent with standard practices in machine learning applications.

### Data sources and study population

The analysis included data from N = 110,251 children aged 6–59 months, of whom 68,566 (62.2%) were classified as anemic based on the WHO hemoglobin thresholds adjusted for age and altitude. To ensure data reliability, the study population was limited to children with complete anthropometric and hemoglobin data. Missing values were imputed using median imputation for continuous variables and mode imputation for categorical variables. Before model development, the final analytic dataset (N = 110,251) was randomly split into a training set (80%, n ≈ 88,201) and a testing set (20%, n ≈ 22,050) using stratified sampling to preserve the outcome distribution.

Ethical approvals for the original DHS data collection were obtained from each country’s relevant ethics committee. This study represents a secondary analysis of anonymized, publicly available DHS data; therefore, no additional institutional ethical approval was required.

Before imputation, a missing profile was generated for all candidate predictors. The proportion of missing values ranged from <1% for child sex and age to approximately 7–8% for vitamin A supplementation and partner education.

### Outcome and predictor variables

We selected variables associated with the risk of childhood anemia based on previous studies [10,11,21,22]. The primary outcome variable was childhood anemia, defined as a binary variable (anemia vs. no anemia) based on hemoglobin concentration adjusted for age and altitude. Variables included in the analysis encompassed individual, maternal, household, and environmental factors relevant to childhood anemia risk. Child-level variables included age in months, sex, stunting (height-for-age z-score), and breastfeeding duration. Maternal factors included age, working status, birth size, iron supplementation during pregnancy, vitamin A intake, and number of living children. Household characteristics included household size, number of children under five, wealth index, partner’s education, type of toilet facility, water source, and residence type. Country of residence was also incorporated to capture regional differences in health infrastructure, nutrition programs, and disease prevalence. These variables reflect established risk factors influencing childhood anemia in Sub-Saharan Africa. The inclusion of country captures contextual heterogeneity (health systems, nutrition programs), but may also act as a proxy variable, which is addressed in the sensitivity analysis.

### Outcome definition

Childhood anemia was defined as hemoglobin concentration <11.0 g/dL for children aged 6–59 months, adjusted for altitude, according to the WHO guidelines [23]. The outcome was coded as a binary variable (anemic vs. non-anemic).

### Data analysis and machine learning modeling

The final analytical dataset included 110,251 children aged 6–59 months. The dataset was randomly split into a training set (80%, n ≈ 88,201) and an independent test set (20%, n ≈ 22,050) using stratified sampling to preserve the class distribution. Hyperparameter tuning was performed within the training set using 5-fold stratified cross-validation, and final model performance was evaluated on the independent test set.

### Data preprocessing and management

Before model development, extensive preprocessing was conducted to handle missing values, normalize the continuous variables, and encode the categorical variables. Missing values for continuous predictors were imputed using predictive mean matching, and categorical variables were imputed using the mode for each stratum of the dataset. Outliers were identified based on z-scores exceeding ±4 and assessed for plausibility; extreme values were retained if they were biologically reasonable.

Continuous variables, including anthropometric z-scores, child age, and household size, were scaled using standardization to a zero mean and unit variance to ensure feature comparability. Categorical variables, such as maternal education level and household wealth index, were transformed into dummy variables for the ML model input. Feature correlation matrices were computed to assess multicollinearity, and highly correlated features (Pearson r > 0.9) were either combined or removed to avoid redundancy in model training. After preprocessing and imputation, the final analytical sample remained n = 110,251 children, which was then split into training (n ≈ 88,201) and testing (n ≈ 22,050) subsets. All preprocessing steps were applied before model training to avoid data leakage.

### Feature selection

This process ensured that only meaningful variables were included in the final models, thereby improving both the prediction performance and interpretability. The selected features included height-for-age z-scores, child age, maternal age, household size, wealth index, partner’s education, birth size, toilet facility type, iron supplementation, vitamin A intake, and country of residence.

### Machine learning model development

Eight machine learning algorithms were applied to predict childhood anemia.

**Random Forest (RF):** An ensemble method that constructs multiple decision trees and aggregates the predictions to reduce overfitting. The tuned hyperparameters included the number of trees (100–500), maximum tree depth, and minimum number of samples per leaf.

**LightGBM:** A gradient boosting framework optimized for speed and memory efficiency, which is particularly effective for high-dimensional datasets. Parameters such as the learning rate, number of leaves, and maximum depth were tuned.

**CatBoost:** A gradient boosting method capable of natively handling categorical variables without extensive preprocessing. Parameters such as the learning rate, depth, and number of iterations were optimized in this study.

**XGBoost:** A highly efficient boosting algorithm that is widely used for tabular data analysis. Hyperparameters, including the learning rate, maximum depth, and number of estimators, were tuned using a randomized search.

**Extra Trees:** An ensemble of randomized decision trees, similar to RF, but with additional randomness in feature splits.

**Logistic Regression:** A traditional linear classifier serving as a baseline. The regularization strength and solver type were tuned.

**Multi-Layer Perceptron (MLP) Neural Network:** A feedforward neural network with one hidden layer; hyperparameters include the number of neurons, learning rate, and activation function.

**Decision Tree:** A single-tree baseline model with depth and minimum samples per leaf tuned to prevent overfitting.

Hyperparameters for each model were optimized using randomized search within the training dataset. Stratified 5-fold cross-validation was applied during hyperparameter tuning to ensure robustness and maintain class distribution. To address class imbalance (62.2% anemia prevalence), class weighting was applied where supported. Threshold tuning was performed using precision–recall curves to optimize the F1-score. Although sensitivity was considered important for public health screening, model evaluation emphasized balanced performance (F1-score and ROC-AUC) due to the observed moderate recall (~0.40).

### Model evaluation metrics

The model performance was evaluated using multiple metrics to capture different aspects of the predictive accuracy. **Accuracy** measured the proportion of correctly classified children; **precision** quantified the proportion of correctly predicted anemic cases among all predicted anemic cases; **recall (sensitivity)** reflected the proportion of true anemic cases correctly identified; the **F1-score** provided a balance between precision and recall; and **ROC-AUC** measured the area under the receiver operating characteristic curve to assess the model’s discriminative ability. Confidence intervals (95%) for ROC-AUC were estimated using bootstrap resampling (500 iterations). Pairwise comparisons between models were conducted using the DeLong test to assess the statistical significance of AUC differences. ROC-AUC and F1-score were prioritized over recall alone, given the moderate sensitivity observed in the final models.

Model calibration was evaluated using calibration curves and Brier scores, and isotonic regression was applied where appropriate. DHS sampling weights (v005), clustering (v021), and stratification variables (v022/v023) were applied in descriptive analyses to ensure representativeness. However, these weights were not incorporated into the machine learning model training due to limitations of standard ML algorithms in handling complex survey designs.

### Software and statistical tools

**Fig 1:**
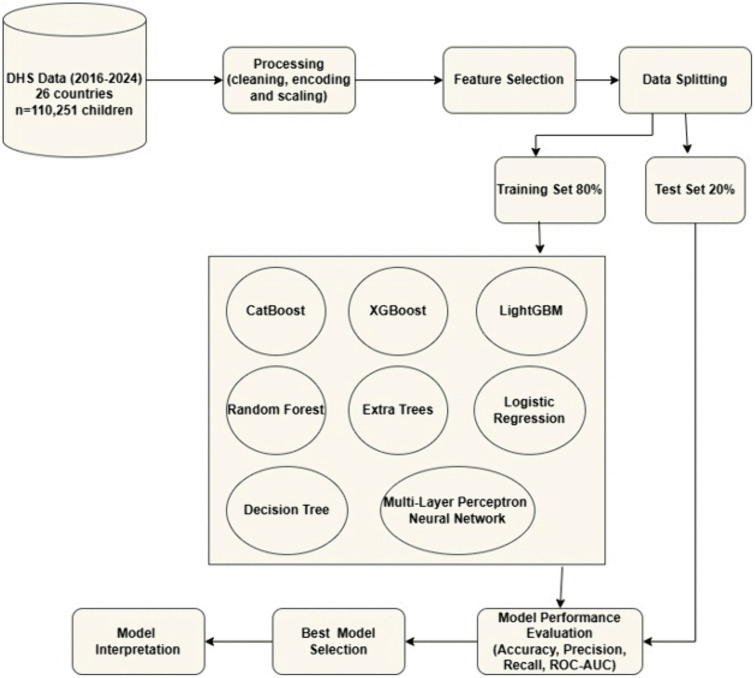
Machine learning workflow for predicting childhood anemia using pooled Demographic and Health Survey (DHS) data from 26 Sub-Saharan African countries (2016–2024).

All analyses were performed in Python 3.12 using libraries including pandas, numpy, scikit-learn, xgboost, lightgbm, catboost, and matplotlib/seaborn for visualization. Hyperparameter tuning was performed using RandomizedSearchCV from scikit-learn. Fig 1 illustrates the overall machine learning pipeline used for predicting childhood anemia using DHS data.

## Results

### Sample characteristics

The study analyzed data from a pooled sample of 110,251 children aged 6–59 months across 26 Sub-Saharan African countries, of whom 68,566 (62.2%) were classified as anemic and 41,685 (37.8%) as non-anemic. A comparative analysis between anemic and nonanemic children revealed notable differences in sociodemographic, environmental, and health-related variables.

The maternal age distribution showed a modest variation between the groups. A slightly higher proportion of mothers aged <20 years was observed among anemic (5.9%) than among nonanemic children (3.4%). Conversely, mothers aged 30–39 years constituted a somewhat smaller proportion of the anemic group (36.6%) than the nonanemic group (39.8%).

Clear socioeconomic disparities were evident in the level of paternal education. Children with anemia were more likely to have fathers with no formal education (38.9% vs. 26.7%), while higher paternal education was less common among anemic children (6.2%) than among nonanemic children (9.2%), suggesting a gradient of educational disadvantage.

Sex distribution was relatively balanced, with male children slightly overrepresented among the anemic cases (51.8%) compared with the nonanemic children (48.7%). Female children accounted for 48.2% of the anemic cases and 51.3% of the nonanemic cases, indicating no major sex-based disparity.

Children aged 6–23 months comprised 42.3% of the anemic cases, compared with 24.5% among nonanemic children. In contrast, older children (24–59 months) were more represented in the non-anemic group (75.5%), highlighting increased vulnerability among younger children.

Environmental characteristics showed modest differences. Access to improved toilet facilities was similar across groups (12.5% among anemic vs. 12.1% among non-anemic children). Improved water sources were slightly less common among anemic children (54.6%) compared with non-anemic children (57.0%), though differences were not substantial.

Child morbidity indicators revealed that recent fever (23.8% vs. 17.0%) and diarrhea (16.9% vs. 12.4%) were more frequently reported among anemic children, suggesting a potential contribution of infectious conditions to anemia risk.

Residence patterns indicated that anemia was more prevalent among rural children (71.1%) compared with non-anemic children (65.5%), reflecting a rural disadvantage. Urban residence was relatively more common among non-anemic children (34.5%).

Wealth index distribution demonstrated a clear socioeconomic gradient. The poorest wealth quintile accounted for 28.8% of anemic children compared with 21.6% of non-anemic children, while the richest quintile was underrepresented among anemic children (12.3% vs. 19.1%). These findings indicate that socioeconomic deprivation remains an important correlate of childhood anemia.

Birth size distribution was largely similar between groups, with average birth size accounting for more than 93% in both categories. Differences in very small or very large birth size were minimal.

Micronutrient supplementation showed modest variation. Vitamin A supplementation was slightly more common among anemic children (37.3%) compared with non-anemic children (31.8%). Iron supplementation coverage was comparable across groups (54.5% among anemic vs. 49.0% among non-anemic), suggesting limited differentiation by anemia status. (Table 1).

**Table 1:** Comparison of Characteristics Between Anemic and Non-Anemic Children Aged 6–59 Months in 26 Sub-Saharan African Countries.

Variable	Level	Anemic % (n = 68566)	Non-anemic % (41685)
Maternal_age_group	<20	5.9	3.4
	20-29	48.2	45.9
	30-39	36.6	39.8
	>40	9.3	10.8
Paternal_education_level	No education	38.9	26.7
	Primary	28.4	32.4
	Secondary	26.5	31.7
	Higher	6.2	9.2
Child_sex	Female	48.2	51.3
	Male	51.8	48.7
Child_age_group	6-23	42.3	24.5
	24-59	57.7	75.5
Toilet_facility	Improved	12.5	12.1
	Non-improved	87.5	87.9
Water_source	Improved	54.6	57.0
	Non-improved	45.4	43.0
Child_fever	Yes	23.8	17.0
	No	76.2	83.0
Child_diarrhea	Yes	16.9	12.4
	No	83.1	87.6
Residence	Rural	71.1	65.5
	Urban	28.9	34.5
Wealth_index	Middle	19.8	20.1
	Poorer	22.4	19.9
	Poorest	28.8	21.6
	Richer	16.7	19.3
	Richest	12.3	19.1
Child_birth_size	Average	93.9	94.1
	Larger than average	2.4	2.5
	Smaller than average	1.0	1.0
	Very large	2.0	1.8
	Very small	0.7	0.6
Vitamin_A	Yes	37.3	31.8
	No	62.7	68.2
Iron_supplement	Yes	54.5	49.0
	No	45.5	51.0

**Fig 2:**
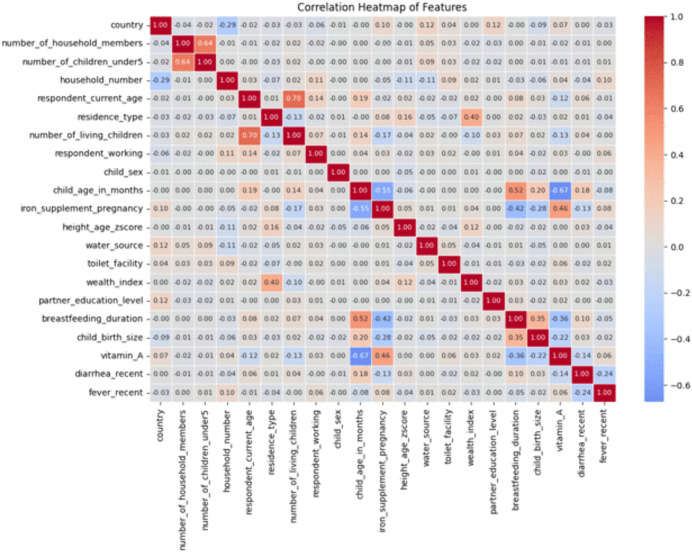
Correlation heatmap of predictor variables included in the machine learning models.

Correlation heatmap illustrating the relationships among multiple features from the dataset. The heatmap uses a color scale from red to blue, where red indicates a strong positive correlation (close to 1.0), blue indicates a strong negative correlation (close to -1.0), and white or light colors indicate weak or no correlation (Fig 2).

### Machine learning model performance

Eight machine learning models were trained and evaluated using stratified 5-fold cross-validation. Table 2 summarizes the performance metrics of accuracy, precision, recall, F1-score, and ROC-AUC.

**Table 2:** Model Performance Comparison in Predicting Childhood Anemia.

Model	Accuracy	Precision	Recall	F1 Score	ROC-AUC	CI 95%
CatBoost	0.795	0.614	0.401	0.485	0.844	0.840-0.848
LightGBM	0.792	0.603	0.407	0.486	0.841	0.837-0.844
XGBoost	0.791	0.595	0.411	0.486	0.839	0.835-0.842
Random Forest	0.788	0.601	0.358	0.449	0.837	0.833-0.840
Extra Trees	0.780	0.575	0.332	0.421	0.822	0.818-0.826
MLP	0.770	0.538	0.328	0.408	0.811	0.807-0.815
Logistic Regression	0.768	0.535	0.276	0.364	0.800	0.795-0.804
Decision Tree	0.731	0.443	0.457	0.450	0.637	0.631-0.643

**CI**: 95% confidence interval for ROC-AUC estimated using bootstrap resampling (500 iterations).

The CatBoost model achieved the highest discriminative performance (AUC = 0.844, 95% CI: 0.840–0.848), followed closely by LightGBM and XGBoost. Although the differences in AUC were statistically significant, the magnitude of improvement was modest, as reflected by overlapping confidence intervals. Tree-based ensemble models consistently outperformed logistic regression in terms of overall discrimination. Notably, the Decision Tree model demonstrated relatively higher recall but substantially lower AUC, indicating poor overall predictive performance (Table 2).

The ROC curves illustrate the comparative performance of eight tuned machine learning models in predicting childhood anemia, with each curve representing the trade-off between sensitivity (true positive rate) and specificity (1 − false positive rate) across probability thresholds. Among the evaluated models, CatBoost demonstrated the highest discriminatory ability, achieving an Area Under the Curve (AUC) of 0.844, indicating the strongest overall capacity to distinguish between anemic and non-anemic children.

**Fig 3:**
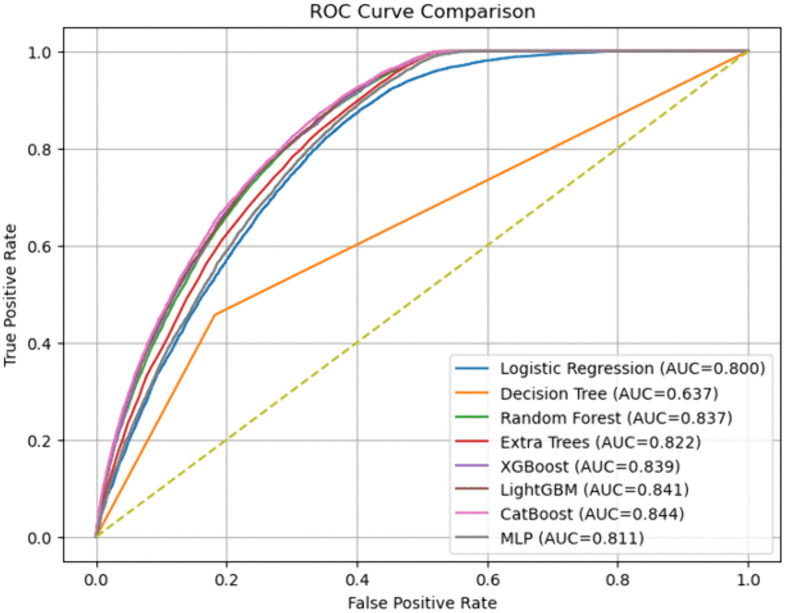
Receiver operating characteristic (ROC) curves comparing the performance of eight machine learning models for predicting childhood anemia.

Gradient boosting methods performed consistently well, with LightGBM (AUC = 0.841) and XGBoost (AUC = 0.839) showing comparable discrimination, closely approaching CatBoost’s performance. The Random Forest model (AUC = 0.837) also demonstrated strong predictive capability, though slightly lower than the boosting-based approaches (Fig 3).

**Fig 4:**
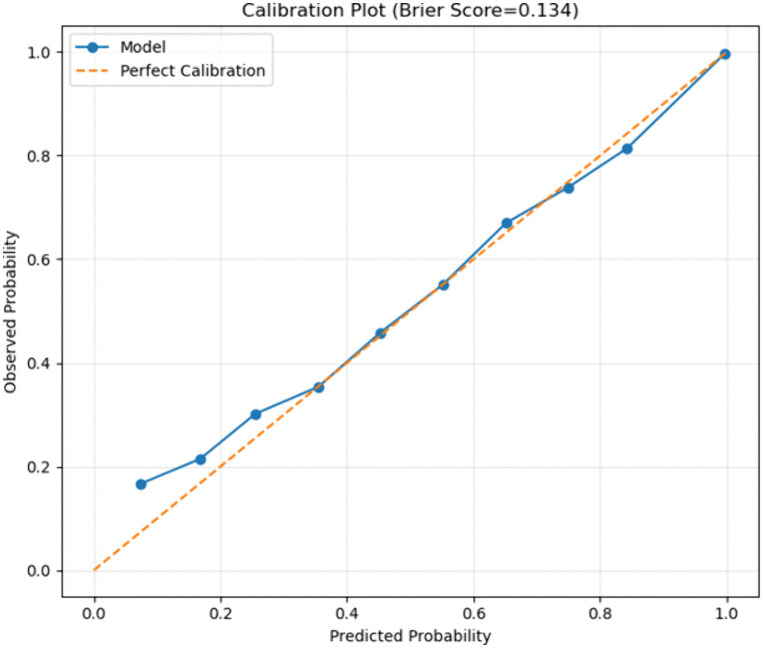
Calibration curve of the CatBoost model.

The provided calibration curve indicates that your CatBoost model is exceptionally well-calibrated for the majority of its prediction range, particularly between 0.0 and 0.8, where the blue line closely follows the dashed identity line. This alignment suggests that the model’s predicted probabilities are highly reliable and can be interpreted as actual risk or likelihood without further adjustment (Fig 4).

**Fig 5:**
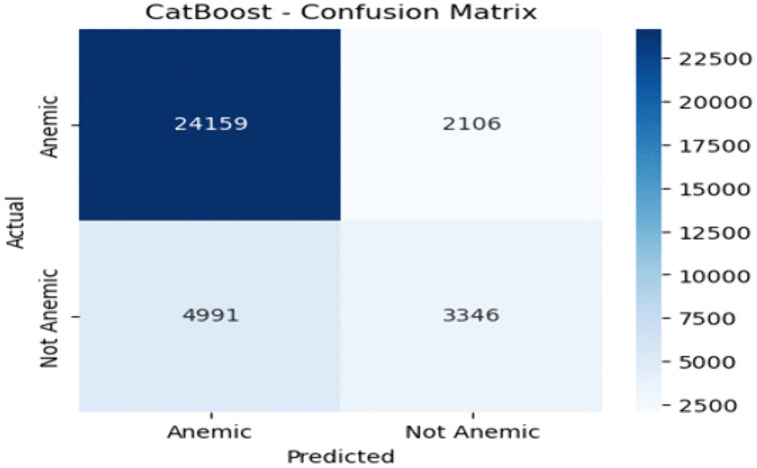
Confusion matrix for the CatBoost model on the independent test dataset.

The confusion matrix illustrates the performance of the CatBoost model in classifying anemia. The model achieved high sensitivity, correctly identifying the majority of anemic children (true positives), while a smaller proportion were misclassified as non-anemic (false negatives). Among non-anemic children, a moderate number were correctly classified (true negatives), although some were incorrectly predicted as anemic (false positives). Overall, the model prioritizes sensitivity, making it suitable for public health screening, albeit with reduced specificity (Fig 5).

### Feature importance analysis

CatBoost feature analysis showed that the following features are the most relevant for predicting childhood anemia among children aged 6–59 months.

The CatBoost model identifies residence type, height-for-age z-score, country context, and child age in months as the most influential predictors of childhood anemia, with maternal characteristics contributing more modestly. These findings indicate that anemia is not solely a biological condition but is strongly shaped by geographic, environmental, and structural predictors.

Child age in months also demonstrates substantial influence, highlighting early childhood, particularly the 6–23 month period, as a critical window of vulnerability. During this stage, iron stores acquired at birth become depleted while rapid growth substantially increases nutritional requirements. Inadequate complementary feeding during this transition further amplifies anemia risk.

Residence type and country-level variation rank among the strongest predictors, underscoring that contextual and structural disparities significantly shape anemia risk. Differences in healthcare access, malaria burden, sanitation infrastructure, food security, and national nutrition policies likely explain much of this heterogeneity.

**Fig 6:**
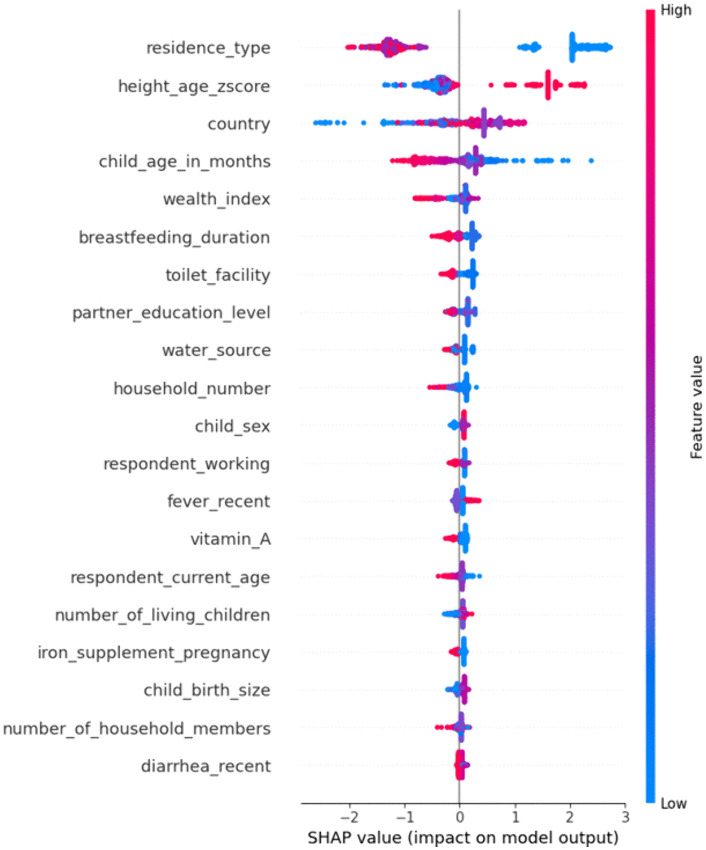
SHAP summary plot showing the relative importance of predictors in the CatBoost model.

Maternal age shows a comparatively smaller but meaningful contribution, suggesting that maternal context influences anemia risk indirectly through caregiving capacity, socioeconomic position, and maternal nutritional status. This points toward an intergenerational dimension of risk, though broader environmental and nutritional factors appear to drive the majority of predictive power in the pooled model. (Fig 6).

**Fig 7:**
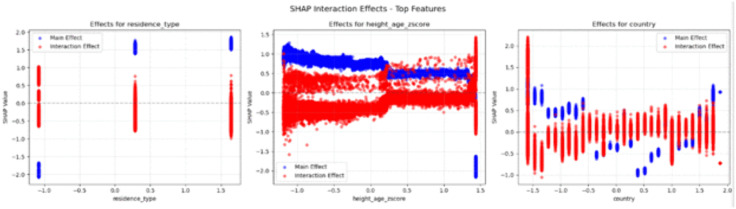
SHAP interaction plots illustrating interactions among key predictors.

The SHAP interaction plots further clarify that the effects of residence type, height-for-age z-score, and country on childhood anemia are not purely independent but are modified by interactions with other variables. Residence type shows a strong main effect, with clear separation between categories, while the interaction values indicate that its impact varies depending on additional contextual and household factors. Height-for-age z-score demonstrates a pronounced main protective effect at higher values (better nutritional status), but the interaction spread suggests that the magnitude of its influence changes across different environments and socioeconomic conditions. The country exhibits substantial variability in both main and interaction effects, confirming that structural, epidemiological, and policy differences across settings shape anemia risk in complex ways. Overall, these interaction patterns reinforce that childhood anemia arises from interconnected nutritional, geographic, and contextual predictors rather than isolated predictors, highlighting the need for multifaceted and country-specific intervention strategies. (Fig 7).

### Country-level insights

The pooled analysis across 26 Sub-Saharan African countries reveals a substantial and widespread burden of childhood anemia, with an overall prevalence of 62.2%. In most countries included in the analysis, more than half of children aged 6–59 months were classified as anemic, highlighting a serious regional public health concern.

The highest prevalence was observed in Mali (80.2%), followed by Mauritania (75.5%), Guinea (74.1%), and Burkina Faso (72.2%). Similarly elevated prevalence rates were recorded in Côte d’Ivoire (71.1%), Liberia (71.1%), Mozambique (69.8%), Sierra Leone (69.7%), Gabon (69.0%), and Nigeria (68.8%). These findings indicate that in several West and Central African countries, nearly two-thirds to four-fifths of children are affected by anemia, posing serious implications for cognitive development, immune function, educational attainment, and long-term productivity.

Moderately high prevalence levels were documented in Angola (64.9%), Benin (65.2%), Malawi (63.1%), South Africa (61.1%), Tanzania (60.6%), Ethiopia (60.2%), Burundi (59.1%), Lesotho (59.0%), and Zambia (58.5%), further demonstrating the extensive regional distribution of anemia.

In contrast, substantially lower prevalence rates were observed in Rwanda (36.6%) and Zimbabwe (38.1%), followed by Madagascar (47.7%), Gambia (53.4%), Ghana (54.2%), and Uganda (54.6%). These comparatively lower rates suggest potential differences in nutrition programs, malaria control strategies, health system performance, socioeconomic conditions, or maternal and child health interventions. (Table 3).

**Table 3:** Childhood Anemia Prevalence among children aged 6-59 months in 26 African Countries.

Country	Not Anemic	Anemic	Total	Prevalence (%)
Angola	1,978	3,657.0	5,635	64.9
Benin	324	608.0	932	65.2
Burkina Faso	1,424	3,699.0	5,123	72.2
Burundi	2,218	3,205.0	5,423	59.1
Cameroon	1,695	2,292.0	3,987	57.5
Cote d’Ivoire	1,187	2,925.0	4,112	71.1
Ethiopia	3,104	4,691.0	7,795	60.2
Gabon	1,442	3,217.0	4,659	69.0
Gambia	1,526	1,748.0	3,274	53.4
Ghana	1,778	2,104.0	3,882	54.2
Guinea	786	2,254.0	3,040	74.1
Lesotho	385	553.0	938	59.0
Liberia	622	1,532.0	2,154	71.1
Madagascar	2,669	2,439.0	5,108	47.7
Malawi	1,726	2,950.0	4,676	63.1
Mali	765	3,108.0	3,873	80.2
Mauritania	1,024	3,152.0	4,176	75.5
Mozambique	1,022	2,366.0	3,388	69.8
Nigeria	3,179	7,003.0	10,182	68.8
Rwanda	2,179	1,257.0	3,436	36.6
Sierra Leone	1,064	2,446.0	3,510	69.7
South Africa	321	504.0	825	61.1
Tanzania	1,686	2,597.0	4,283	60.6
Uganda	1,792	2,152.0	3,944	54.6
Zambia	3,217	4,526.0	7,743	58.5
Zimbabwe	2,572	1,581.0	4,153	38.1
Total	**41,685**	**68,566.0**	**110,251**	**62.2**

## Discussion

This study developed and compared multiple machine learning models for predicting childhood anemia using large-scale DHS data across Sub-Saharan Africa. The CatBoost model achieved the highest performance (AUC ≈ 0.84), indicating moderate to strong discrimination. Among the evaluated algorithms, CatBoost demonstrated the strongest overall discriminatory performance, while LightGBM and XGBoost performed comparably. The findings are consistent with prior studies showing improved predictive performance of ensemble models over traditional regression approaches. Reinforcing prior evidence that boosting algorithms are particularly effective for high-dimensional tabular health data [12,14]. These findings support the suitability of advanced ML approaches for modeling complex, nonlinear relationships within multi-country population datasets.

Although boosting models achieved strong overall discrimination, recall values remained moderate across algorithms. This suggests that while ML models can meaningfully stratify anemia risk at the population level, further threshold optimization may be required for high-sensitivity screening purposes. Nonetheless, ML-based risk prediction offers advantages over conventional regression in capturing nonlinear interactions and high-order feature dependencies [15].

Feature importance and SHAP analyses identified stunting (height-for-age z-score) as the most influential predictor of childhood anemia. This aligns with established evidence linking chronic undernutrition to impaired hemoglobin synthesis and long-term developmental consequences [18,19]. Undernutrition remains a key driver of anemia in low- and middle-income countries, particularly in SSA [3,5]. The strong contribution of anthropometric status underscores the biological interconnection between chronic nutritional deprivation and hematologic outcomes.

Child age emerged as another critical determinant, with children aged 6–23 months demonstrating substantially higher predicted risk. This period corresponds to declining neonatal iron stores and increased physiological demands during rapid growth, combined with suboptimal complementary feeding practices [19]. Previous multi-country analyses have similarly documented elevated anemia prevalence among younger children in SSA and South Asia [11,22].

Maternal age demonstrated a nonlinear association, with children of very young and older mothers exhibiting higher predicted risk. This pattern is consistent with evidence linking maternal nutritional status and reproductive factors to childhood anemia and adverse health outcomes [24,25]. These findings highlight the intergenerational dimension of anemia risk, whereby maternal health, education, and caregiving capacity influence child nutritional outcomes.

Household and socioeconomic factors also contributed substantially to the prediction. Larger household size and a greater number of under-five children were associated with increased anemia risk, likely reflecting constrained household resources and reduced dietary adequacy. Conversely, higher wealth index and partner’s education were protective, consistent with prior DHS-based studies demonstrating socioeconomic gradients in childhood anemia [10,11]. Access to improved sanitation and safe water was similarly associated with reduced risk, likely mediated through decreased infection burden and improved environmental health [26].

Notably, country of residence contributed meaningfully to prediction, underscoring the role of contextual predictors such as malaria prevalence, food security, national nutrition programs, and health system performance. Substantial cross-country heterogeneity in anemia burden has been widely documented in global estimates [5,6], reinforcing the need for country-specific strategies.

### Policy implications

These findings suggest that machine learning models can support population-level targeting of high-risk groups [7,27]. Second, enhancing maternal health services, particularly antenatal care and iron supplementation during pregnancy, may mitigate intergenerational transmission of anemia risk [24]. Third, structural interventions addressing poverty, sanitation, and educational attainment are essential components of comprehensive anemia reduction strategies [3]. Given the observed country-level variation, interventions should be tailored to national epidemiological contexts rather than implemented uniformly across the region.

ML-based risk prediction models may complement existing public health programs by identifying high-risk subgroups and informing targeted resource allocation, especially within digital health and child nutrition monitoring platforms [27].

Future research should extend beyond predictive modeling to examine causal pathways linking nutritional, maternal, household, and environmental predictors to childhood anemia. While this study demonstrated the utility of ML methods, additional work integrating biomarkers of iron, folate, and vitamin B12 deficiency would improve specificity in identifying etiological subtypes of anemia [6,7]. Longitudinal studies are needed to evaluate the temporal impact of maternal health interventions, household food security, and sanitation improvements on anemia outcomes. Moreover, implementation research should assess how ML-based risk prediction tools can be embedded into national health information systems, as piloted in digital health platforms for child nutrition monitoring [27]. Multi-country collaborations combining survey, clinical, and geospatial data would further enhance predictive accuracy and guide regionally tailored interventions.

### Strengths and limitations of the study

This study has several notable strengths. The use of large, nationally representative DHS datasets from 26 Sub-Saharan African countries enhances external validity and supports broad generalizability across heterogeneous epidemiological and socioeconomic contexts. The substantial pooled sample (n = 110,251) provided strong statistical power and enabled robust cross-country comparisons. A comprehensive evaluation of eight machine learning algorithms, including multiple ensemble and gradient boosting methods, allowed rigorous benchmarking of predictive performance. Furthermore, SHAP-based interpretability strengthened model transparency, facilitating identification of stable, biologically plausible, and policy-relevant predictors. Evaluation of both discrimination and calibration further enhanced methodological robustness.

Several limitations warrant consideration. The cross-sectional design precludes causal inference and limits assessment of temporal relationships. Key etiological factors such as detailed dietary intake, micronutrient biomarkers, malaria parasitemia, helminth infection, and hemoglobinopathies were unavailable or incompletely captured, constraining etiological specificity. Although discrimination was strong, model sensitivity remained moderate, potentially limiting immediate application as a stand-alone screening tool without threshold optimization. Additionally, machine learning algorithms do not fully account for complex survey design in the same manner as traditional weighted regression models, which may introduce minor bias. Finally, cross-country heterogeneity in survey timing and health system contexts may contribute to variability in predictive performance, although inclusion of country-level effects partially mitigates this concern.

## Conclusion

Childhood anemia remains highly prevalent across Sub-Saharan Africa, affecting over 60% of children aged 6–59 months in this pooled analysis. Ensemble machine learning models, particularly CatBoost, demonstrated strong discriminatory performance in predicting anemia risk across diverse national contexts. Key predictors included chronic undernutrition, early childhood age, and contextual socioeconomic factors. These findings highlight the potential of interpretable machine learning to support targeted screening and precision public health strategies. Strengthening early-life nutrition and maternal health interventions remains critical to reducing the regional anemia burden.

## References

[1] McLean E, Cogswell M, Egli I, Wojdyla D, De Benoist B. Worldwide prevalence of anaemia, WHO vitamin and mineral nutrition information system, 1993–2005. Public Health Nutr. 2009;12(4):444–54. 18498676 10.1017/S1368980008002401

[2] Organization WH. Anaemia fact sheet. 2024.

[3] Balarajan Y, Ramakrishnan U, Ozaltin E, Shankar AH, Subramanian SV. Anaemia in low-income and middle-income countries. Lancet. 2011;378(9809):2123–35. doi: 10.1016/S0140-6736(10)62304-5 21813172

[4] Black RE, Allen LH, Bhutta ZA, Caulfield LE, de Onis M, Ezzati M, et al. Maternal and child undernutrition: global and regional exposures and health consequences. Lancet. 2008;371(9608):243–60. doi: 10.1016/S0140-6736(07)61690-0 18207566

[5] Stevens GA, Finucane MM, De-Regil LM, Paciorek CJ, Flaxman SR, Branca F, et al. Global, regional, and national trends in haemoglobin concentration and prevalence of total and severe anaemia in children and pregnant and non-pregnant women for 1995-2011: a systematic analysis of population-representative data. Lancet Glob Health. 2013;1(1):e16-25. doi: 10.1016/S2214-109X(13)70001-9 25103581 PMC4547326

[6] Kassebaum NJ, Jasrasaria R, Naghavi M, Wulf SK, Johns N, Lozano R, et al. A systematic analysis of global anemia burden from 1990 to 2010. Blood. 2014;123(5):615–24. doi: 10.1182/blood-2013-06-508325 24297872 PMC3907750

[7] Pasricha S-R, Drakesmith H, Black J, Hipgrave D, Biggs B-A. Control of iron deficiency anemia in low- and middle-income countries. Blood. 2013;121(14):2607–17. doi: 10.1182/blood-2012-09-453522 23355536

[8] Rahman MM, Abe SK, Rahman MS, Kanda M, Narita S, Bilano V, et al. Maternal anemia and risk of adverse birth and health outcomes in low- and middle-income countries: systematic review and meta-analysis. Am J Clin Nutr. 2016;103(2):495–504. doi: 10.3945/ajcn.115.107896 26739036

[9] Rai A, Khan MN, Thapa S. Trends and determinants of anaemia in women of Nepal: a multilevel analysis. Matern Child Nutr. 2020;16(4):e13044. doi: 10.1111/mcn.13044 32627381 PMC7507699

[10] Jember TA, Teshome DF, Gezie LD, Agegnehu CD. Spatial variation and determinants of childhood anemia among children aged 6 to 59 months in Ethiopia: further analysis of Ethiopian demographic and health survey 2016. BMC Pediatr. 2021;21(1):497. doi: 10.1186/s12887-021-02901-y 34753442 PMC8576906

[11] Chowdhury MRK, Khan MMH, Khan HTA, Rahman MS, Islam MR, Islam MM, et al. Prevalence and risk factors of childhood anemia in Nepal: A multilevel analysis. PLoS One. 2020;15(10):e0239409. doi: 10.1371/journal.pone.0239409 33021981 PMC7537867

[12] Chen T, Guestrin C. Xgboost: A scalable tree boosting system. In: Proceedings of the 22nd ACM SIGKDD International Conference on Knowledge Discovery and Data Mining. 2016.

[13] Ke G, Meng Q, Finley T, Wang T, Chen W, Ma W, et al. Lightgbm: A highly efficient gradient boosting decision tree. Adv Neural Inform Process Syst. 2017;30.

[14] Prokhorenkova L, Gusev G, Vorobev A, Dorogush AV, Gulin A. CatBoost: unbiased boosting with categorical features. Adv Neural Inform Process Syst. 2018;31.

[15] Lundberg SM, Lee SI. A unified approach to interpreting model predictions. Adv Neural Inform Process Syst. 2017;30.

[16] Kursa MB, Rudnicki WR. Feature Selection with theBorutaPackage. J Stat Soft. 2010;36(11). doi: 10.18637/jss.v036.i11

[17] UNICEF. Levels and trends in child anemia. New York: UNICEF; 2020.

[18] Dewey KG, Begum K. Long-term consequences of stunting in early life. Matern Child Nutr. 2011;7(Suppl 3):5–18. doi: 10.1111/j.1740-8709.2011.00349.x 21929633 PMC6860846

[19] Black RE, Bhutta ZA, Alderman H, Ruel MT, Gillespie S, Haddad LJ. Maternal and child nutrition–Authors’ reply. 2013. 10.1016/S0140-6736(13)62320-X 24209822

[20] United Nations Children’s F, World Health O, World B. Levels and trends in child malnutrition: UNICEF / WHO / The World Bank Group joint child malnutrition estimates: key findings of the 2020 edition. Geneva: World Health Organization; 2020.

[21] Abdullah M, Al-Asmari S. Anemia types prediction based on data mining classification algorithms. Communication, management and information technology. CRC Press; 2016. p. 629–36.

[22] Khan JR, Awan N, Misu F. Determinants of anemia among 6-59 months aged children in Bangladesh: evidence from nationally representative data. BMC Pediatr. 2016;16:3. doi: 10.1186/s12887-015-0536-z 26754288 PMC4707771

[23] World Health Organization. Haemoglobin concentrations for the diagnosis of anaemia and assessment of severity. World Health Organization; 2011.

[24] Rahman MM, Abe SK, Rahman MS, Kanda M, Narita S, Bilano V, et al. Maternal anemia and risk of adverse birth and health outcomes in low-and middle-income countries: systematic review and meta-analysis, 2. The Am J Clin Nutrit. 2016;103(2):495–504. 26739036 10.3945/ajcn.115.107896

[25] Rai A, Khan MN, Thapa S. Trends and determinants of anaemia in women of Nepal: a multilevel analysis. Matern Child Nutr. 2020;16(4):e13044. doi: 10.1111/mcn.13044 32627381 PMC7507699

[26] Organization WH. Anaemia fact sheet. 2024.

[27] UNICEF. UNICEF nutrition strategy 2020–2030: Nutrition for every child. United Nations Children’s Fund. 2020.

